# Pomegranate Peel-Derived Hard Carbons as Anode Materials for Sodium-Ion Batteries

**DOI:** 10.3390/molecules29194639

**Published:** 2024-09-29

**Authors:** Qijie Wu, Kewei Shu, Long Zhao, Jianming Zhang

**Affiliations:** 1 Institute of Quantum and Sustainable Technology (IQST), School of Chemistry and Chemical Engineering, Jiangsu University, Zhenjiang 212013, China; qw988@ujs.edu.cn (Q.W.); longzhao@ujs.edu.cn (L.Z.); 2 College of Chemistry and Chemical Engineering, Shaanxi University of Science and Technology, Xi’an 710021, China

**Keywords:** hard carbon, carbon anode, sodium-ion battery, carbonization, biowaste

## Abstract

Exploring high-performance carbon anodes that are low-cost and easily accessible is the key to the commercialization of sodium-ion batteries. Producing carbon materials from bio by-products is an intriguing strategy for sodium-ion battery anode manufacture and for high-value utilization of biomass. Herein, a novel hard carbon (PPHC) was prepared via a facile pyrolysis process followed by acid treatment using biowaste pomegranate peel as the precursor. The morphology and structure of the PPHC were influenced by the carbonization temperature, as evidenced by physicochemical characterization. The PPHC pyrolyzed at 1100 °C showed expanded interlayer spacing and appropriate oxygen group content. When used as a sodium ion battery anode, the PPHC-1100 demonstrated a reversible capacity of up to 330 mAh g^−1^, maintaining 174 mAh g^−1^ at an increased current rate of 1 C. After 200 cycles at 0.5 C, the capacity delivered by PPHC-1100 was 175 mAh g^−1^. The electrochemical behavior of PPHC electrodes was investigated, revealing that the PPHC-1100 possessed increased capacitive-controlled energy storage and improved ion transport properties, which explained its excellent electrochemical performance. This work underscores the feasibility of high-performance sodium-ion battery anodes derived from biowaste and provides insights into the sodium storage process in biomass-derived hard carbon.

## 1. Introduction

Sodium-ion batteries (SIBs) have attracted tremendous attention in recent years, due to the growing demand for secondary batteries in portable electronics, electric vehicles, and grid storage [1,2,3,4]. SIBs offer several advantages, such as low cost, easy manufacturing, and a wide operation temperature range, making them a viable alternative or complementary technology to lithium-ion batteries (LIBs) [5]. The key challenge for the large-scale production and commercialization of SIBs is to seek suitable electrode materials [6]. Anode materials for SIBs can be classified into alloy type, conversion type, and intercalation type based on their electrochemical mechanisms [7,8]. Among the various anode materials, carbon materials are particularly notable for their light weight, easy accessibility, and stable electrochemical properties [9]. However, the difference in the radius of charge carrier ions leads to distinct design strategies of carbon anodes for SIBs compared to those for LIBs. Graphitic carbon has demonstrated poor electrochemical performance, with a very limited capacity of less than 150 mAh g^−1^ [10]. Therefore, non-graphitic carbon, including hard carbon and soft carbon, is considered an ideal choice for SIB anodes.

Hard carbon is a class of disordered, amorphous carbon which is composed of ordered short-range stacked graphene layers but exhibits long-range disordering [11]. Hard carbon can be easily prepared by pyrolysis of carbon precursors such as natural or synthetic organic polymers, fossil resources, and biomass [12,13,14]. Glucose-based hard carbon was first developed in 2000, showing a reversible capacity of 300 mAh g^−1^ with a low sodium intercalation potential of 0.2 V [15]. Later on, many natural and synthetic polymers including cellulose, phenolic resin [16], polyvinylpyrrolidone [17], and polyacrylonitrile [18] were used as precursors for HC, offering excellent structural flexibility and purity [19]. However, production using those precursors also suffers from relatively high costs. Biomass, especially waste and by-products, is a more promising carbon source due to its unique physicochemical properties, wide distribution, natural diversity, and environmental friendliness [20]. Agricultural by-product rice husk was used to prepare hard carbon via direct pyrolysis at a specific temperature. The rice-husk derived carbon (RHHC) exhibited a large reversible capacity of 372 mAh g^−1^ with an initial coulombic efficiency (ICE) of 66% and good cycle performance. The RHHC-based full cell achieved a high-energy density of 185 Wh kg^−1^ [21]. Multichannel structured hard carbon was obtained via simple carbonization of golden berry leaves at temperatures ranging from 1000 °C to 2000 °C [22]. When carbonized at 1400 °C, the leaf-derived hard carbon displayed optimal electrochemical performance with a reversible capacity of 338.7 mAh g^−1^ and an ICE of 86.43%. The ratio of plateau capacity can be adjusted to over 90% by a temperature-regulated strategy. Lignocellulosic waste corncob was utilized as a carbon precursor for the hard carbon anode and as a cellulose source for the battery binder [23]. The corncob-derived carbon anode, combined with a corncob-derived cellulose binder, delivered a capacity of 264 mAh g^−1^ at 1 C (300 mA g^−1^) with excellent retention (84% after 100 cycles). A 2D biomass-based carbon sheet was prepared by high-temperature pretreatment, achieved a specific capacity of 174 mAh g^−1^ at 100 mA g^−1^ with excellent cycle stability after 100 cycles [24].

Pomegranate peel (PP) is a by-product of the fruit juice processing industry, accounting for 30–50% of the weight of the raw pomegranate. Current recycling technologies for PP focus primarily on the extraction of the bioactive species, leaving the majority of the fiber component to be disposed of [25,26]. PP possesses a naturally formed unique hierarchical porous structure, high carbon content, low mineral content, and contains 40–50% of lignocellulosic matter, which makes it suitable as a precursor for many carbonaceous materials. Several researchers have prepared PP-derived activated carbon and utilized it as a highly effective adsorbent for gas, dyes, and pharmaceutical contaminants [27,28,29]. However, few studies have focused on the application of PP-derived carbon in energy-related fields. Heteroatom-doped porous carbon materials were prepared via pyrolysis and activation of PP precursor, demonstrating excellent oxygen reduction activity [30] or supercapacitor performance [31]. In this study, PP-derived hard carbon (PPHC) was prepared by direct pyrolysis followed by acid pretreatment. The correlation between carbonization temperature and the structure of hard carbon was investigated. The PPHC electrode exhibited excellent electrochemical performance as a sodium-ion battery anode, with a highest capacity of 330 mAh g^−1^ at 0.1 C. It also presented good rate capability and cycling stability, maintaining 175 mAh g^−1^ after 200 cycles.

## 2. Results and Discussion

**Figure 1:**
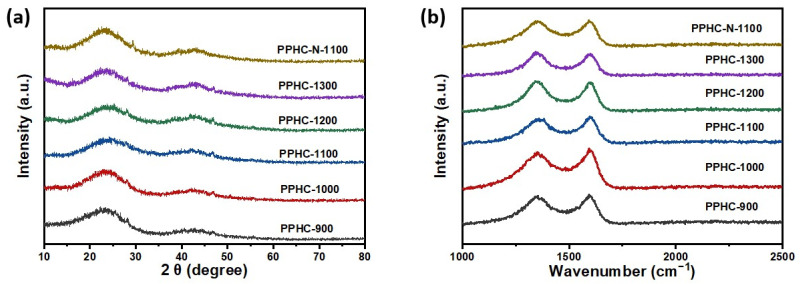
(**a**) XRD patterns; (**b**) Raman spectra of PPHC obtained at different temperatures.

The crystalline structure of the hard carbon was examined by XRD (Figure 1a). Two characteristic peaks of carbon materials appeared at around 22–24° and 44°, corresponding to (002) and (100) planes of graphite, respectively. The small peaks around 28.4° and 47.3° observed in all samples can be attributed to silicon (Si, syn, 27-1402), likely due to equipment or substrate background. All PPHC electrodes exhibit weak and broad (002) peak, indexing their amorphous characteristic. The (002) peak positions of PPHC-900, PPHC-1000, PPHC-1100, PPHC-1200, and PPHC-1300 were observed between 23.1° and 23.6°. The interlayer spacing calculated by Bragg’s equation ranged from 0.37 to 0.38 nm for PPHC, which is much larger than that of graphite. The slight decrease in interlayer spacing as well as the narrowing of the (002) peak (indexed by reduced full width at half maximum) can still be observed as the carbonization temperature increases. The expanded interlayer spacing benefits Na^+^ ion intercalation, thus leading to enhanced capacity, especially in the low-voltage plateau region [32]. It is important to note that larger interlayer spacing does not always correlate with higher capacity once it exceeds the theoretical minimum value for Na^+^ intercalation (0.37 nm) [33]. Raman spectra were recorded to study the electronic structure of PPHC samples (Figure 1b). The peak at around 1350 cm^−1^ and 1598 cm^−1^ can be further fitted to four components (Figure S1) [34,35]. The characteristic D band originates from the breathing mode of sp2 clusters, which becomes active in the presence of disorder, and the G band, assigned to the in-plane vibrations of sp2-bonded carbons, appears at ~1350 cm^−1^ and 1600 cm^−1^ [36,37]. The component at ~1240–1260 cm^−1^, defined as I peak, is attributed to the disordered graphitic lattice (A1g symmetry), polyene-like structure, or ionic impurities. The A peak at ~1500–1520 cm^−1^ is originated from the amorphous carbon or functional residual group (organic molecules, fragments, etc.). The intensity ratio I_D_/I_G_ is an important index to reflect the level of defect and disorder. The area intensity ratio I_D_/I_G_ of the PPHC decreases with the elevated temperature. The I_D_/I_G_ is 1.76 for PPHC-900, then decreases to 1.71, 1.43 for PPHC-1100, PPHC-1300, respectively. The degree of disorder changes slightly as the temperature increases from 900 to 1100 °C, as indicated by stable I_D_/I_G_ ratios and A peak content. The structure becomes more ordered when the carbonization temperature is above 1200 °C, as seen by the increase in the G peak content and the reduction in defects (decreasing D and A peak content), indicating improved graphitic ordering.

**Figure 2:**
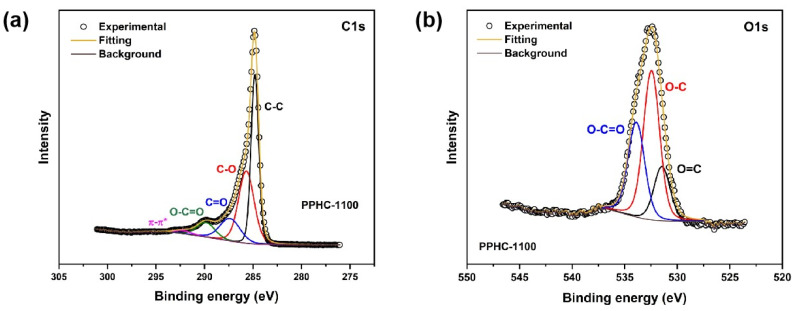
The XPS C1s spectra (**a**) and O1s spectra (**b**) of PPHC-1100.

XPS measurements were conducted to understand the chemical bonding status of the PPHC samples. Emphasis is placed on the oxygen functional group in PPHC, since these groups in carbon materials play a vital role in sodium storage [38,39]. Obvious O peaks appear at ~532 eV (O1s), 980 eV (OKLL)can be observed in the survey scan of the XPS of all PPHC samples (Figure S2). The signals from Ca (330 eV/Ca2p, 450 eV/Ca1s), F (690 eV/F1s, 830 eV/FKLL), and Si (150 eV/Si2s) originate from inorganic impurities, with the total weight ratio for elements other than C and O being less than 5%. The oxygen content for PPHC-900, PPHC-1000, PPHC-1100, PPHC-1200, and PPHC-1300 was 12.3 at%, 10.1 at%, 9.7 at%, 12.0 at%, and 10.1 at%, respectively. The introduction or maintenance of oxygen species can be better tuned at appropriate carbonization temperatures. The C1s peak in the detailed scan can be deconvoluted into four components, located at ~284.8 eV, ~285.7 eV, ~286.9–287.4 eV, 289.7 eV, and 291–292 eV, assigned to (C-C), (C-O), (C=O), (O-C=O), and (π-π*) respectively [40,41,42] (Figure 2a and Figure S3). The O1s spectra further confirm the composition of oxygen-containing species in the hard carbon materials. The peak at ~532 eV can be divided into three components, related to oxygen in carbon–oxygen double bonds (O=C at 531.6 eV), in carbon–oxygen single bonds (O-C at 532.5 eV), and in carboxylic groups (O-C=O at 533.9 eV), respectively [32] (Figure 2b and Figure S4). The existence of abundant oxygen functional groups has multiple impacts on the electrochemical performance of hard carbon materials, including but not limited to expanding the interlayer spacing, inducing additional active sites and improving the electrode/electrolyte interfacial compatibility [43]. The C=O bonds can form stable coordination with Na^+^ to enhance sodium storage via the electrochemical reaction (C=O + Na^+^ + e^−^ → C-O-Na) [44,45]. Theoretical studies have revealed that the C=O in quinones and carboxylic anhydride groups is superior for sodium storage compared to that in carboxylic acid groups [44]. Both C-O and C=O can increase adsorption capacity; however, the former component may result in larger irreversible capacitance [46].

**Figure 3:**
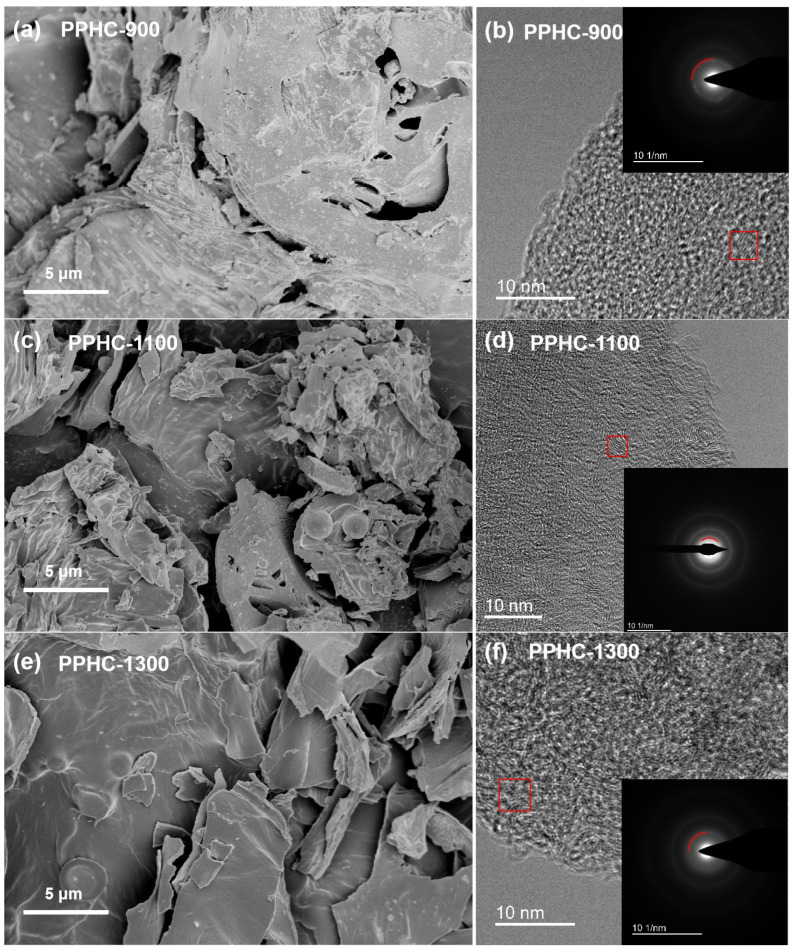
SEM images of PPHC-900 (**a**), PPHC-1100 (**c**), and PPHC-1300 (**e**); and TEM images of PPHC-900 (**b**), PPHC-1100 (**d**), and PPHC-1300 (**f**) (inset: SAED pattern).

Figure 3 presents the SEM images of PPHC obtained at different temperatures. When pyrolyzed at the lower temperature of 900 °C, the characteristic peel structure was partially maintained in PPHC-900 (Figure 3a). The large particle size and relatively low carbonization degree are unfavorable for the sodium storage performance of the hard carbon [47]. At the higher temperature of 1100 °C, most of the biomass structure was decomposed and new particles ranging from 5 to 10 μm were reconstructed. Several wrinkles and folds, as well as corresponding pores with sub-micrometer size, can also be observed in the PPHC-1100 particles (Figure 3c). Such a corrugated surface and porous structure are conducive to sodium-ion diffusion, thus leading to improved electrochemical performance [48,49]. The PPHC-1200 presented a trend of graphitization and loss of surface structure (Figure S5). In the case of PPHC-1300, a graphite-like sheet structure with a smooth surface was observed (Figure 3e). It is well known that carbon with a higher degree of graphitic level presents poor electrochemical performance in sodium-ion batteries [50]. The morphology of PPHC pyrolyzed at 1100 °C (PPHC-N-1100) without acid pretreatment was also compared, showing no significant difference from PPHC-1100 (Figure S6). All PPHC samples have low surface area, for example, 1.7 m^2^ g^−1^ for PPHC-1100, due to a slow heating ramp rate (Figure S7). Although the ideal specific surface area (SSA) for sodium-ion applications has yet to be clearly defined, lower SSA values have a positive impact on battery performance, particularly by reducing electrolyte decomposition and suppressing irreversible capacity loss [39,51,52]. There appears to be no correlation between the trend of specific surface area and capacity. This may be due to the presence of closed pores and ultramicropores, which are undetectable using N_2_ adsorption isotherms. Additionally, previous studies suggest that capacity, particularly the plateau capacity, is associated with the insertion of Na^+^ in graphitic nanodomains rather than the adsorption of Na^+^ in nanopores [39]. TEM images revealed the microstructure of the PPHC materials. All PPHC samples exhibit a turbostratic structure with an occasionally visible layered structure. The SAED pattern of the PPHC samples displayed blurred diffraction rings, indicating the non-graphitic feature of the carbon materials. The interlayer spacing obtained by SAED for PPHC-900, PPHC-1100, and PPHC-1300 was 0.338, 0.368, and 0.352 nm, respectively (Figure 3b,d,f). The expansion of the interlayer spacing can be ascribed to heteroatom doping by bioorganic species in the peel at appropriate temperatures. The amorphous structure of the PPHC can also be evidenced by the faint diffraction rings, corresponding to the (002) and (100) crystalline planes of graphite.

**Figure 4:**
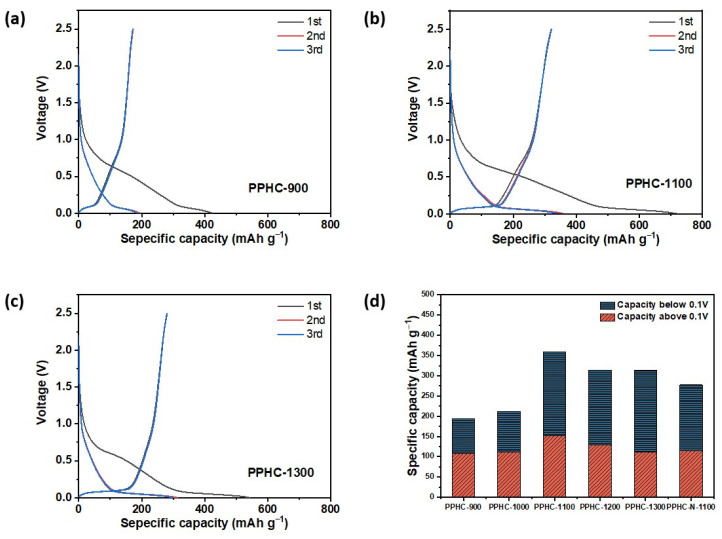
The first three charge–discharge cycles of (**a**) PPHC-900, (**b**) PPHC-1100, (**c**) PPHC-1300 obtained at 0.1 C. (**d**) Specific capacity distribution of PPHC electrodes below and above 0.1 V.

The initial three charge–discharge cycles of PPHC electrodes are presented in Figure 4 and Figure S8. All hard carbon electrodes had a voltage plateau at around 0.5 V during the first discharge, contributing ~100 mAh g^−1^ capacity. This plateau disappeared from the second cycle onwards. This irreversible capacity is due to the formation of solid electrolyte interphase (SEI), which is one of the pronounced features of hard carbon electrodes [53]. The initial coulombic efficiency (ICE) for PPHC-900, PPHC-1000, PPHC-1100, PPHC-1200, and PPHC-1300 was 40.8%, 51.8%, 44.8%, 52.8%, and 51.6%, respectively. The ICE was improved by increasing the carbonization temperature [47]. The coulombic efficiency increased to 86–90% from the second cycle and reached over 90% from the third cycle for all electrodes. The reversible capacity was only 195 mAh g^−1^ for PPHC-900 and was significantly enhanced when the carbonization temperature increased to over 1100 °C. The capacity for PPHC-1100, PPHC-1200, and PPHC-1300 was 359, 314, and 313 mAh g^−1^, respectively. Due to impurities, PPHC-N-1100 provided a capacity of only 277 mAh g^−1^. The whole discharge curve can be divided into two stages: the slope region between 0.1 and 1.0 V and the plateau region below 0.1 V. The former is due to the nanopore filling or Na^+^ intercalation into graphene layers, while the latter can be explained by the binding of Na^+^ at defect sites [15,54]. The slope region capacity accounted for the majority of PPHC-900. The plateau capacity increased significantly from 101 to 207 mAh g^−1^ when the carbonization temperature was higher than 1000 °C, possibly caused by nanopore formation and enlarged interlayer spacing. PPHC-1300 demonstrated decreased slope capacity due to the loss of surface structure at higher temperatures, as revealed by SEM. Of note is the fact that PPHC-1100 not only had a large plateau capacity, but also the highest slope capacity among PPHC electrodes. At intermediate carbonization temperatures, a balance between nanopore formation and oxygen-containing species content was reached.

**Figure 5:**
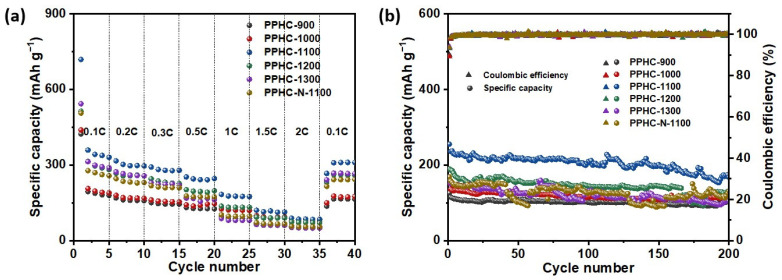
Rate performance (**a**) and cycling stability (**b**) of PPHC electrodes.

The rate performance of the PPHC electrodes was obtained at current densities of 0.1, 0.2, 0.3, 0.5, 1, 1.5, and 2 C (1 C = 300 mAh g^−1^) (Figure 5 and Figure S9). PPHC-1100 exhibited the best comprehensive performance balancing high capacity and rate capability. The capacities of PPHC-1100 at 0.1, 0.2, 0.5, 1, and 2 C were 330, 296, 246, 174, and 84 mAh g^−1^, respectively. The PPHC obtained at lower carbonization temperatures (<1200 °C) showed better rate performance, while PPHC-1300 displayed the lowest retention of 28% (capacity at 1 C/capacity at 0.1 C). PPHC-1200 and PPHC-1100 demonstrated a better balance between capacity and rate performance. The retention of PPHC-1100 at 1 C was 53% of its initial value at 0.1 C. As previously mentioned, slope capacity is related to the surface electrochemical reaction of Na^+^ ions (e.g., reaction with C=O/O-H). Therefore, PPHC electrodes with a higher ratio of slope capacity showed improved rate performance. The cycle performance of PPHC electrodes was obtained at 0.5 C over 200 cycles. PPHC-900 presented a higher capacity retention of 81% but provided a low capacity of 126 mAh g^−1^. PPHC-1000, PPHC-1100, PPHC-1200, and PPHC-1300 had capacity retentions of 71%, 68%, 70%, and 66%, respectively. Although PPHC-1100 did not have an advantage in capacity retention, it provided a larger capacity of 255 mAh g^−1^ (and 175 mAh g^−1^ after 200 cycles). A previous study revealed that a ~30% volume expansion for sodiation of hard carbon [55]. Our post-characterization also observed evidence of volume expansion (Figure S10). A higher degree of graphitization favors the plateau region capacity, which significantly contributes to the capacity loss during cycling [56]. The low retention of PPHC-1300 can be attributed to its increased graphitization. A comparison table demonstrates that our sample has good performance (Table S1).

**Figure 6:**
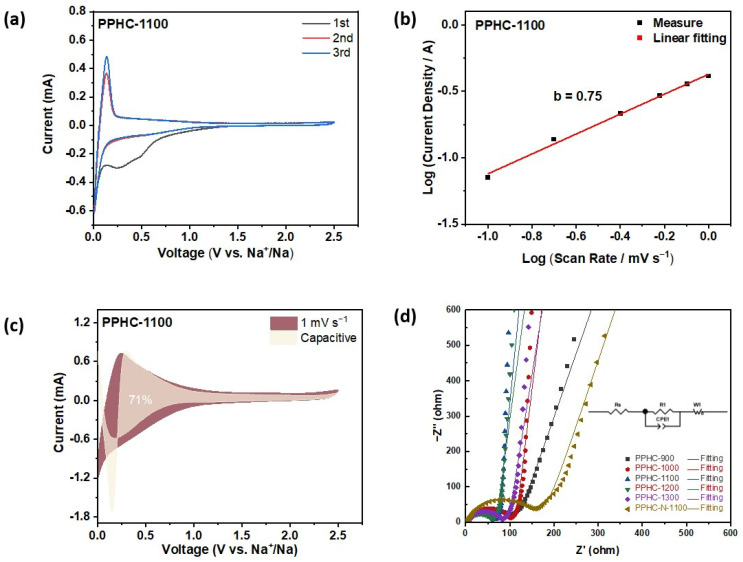
(**a**) CV curves of PPHC-1100 at 0.1 mV s^−1^; (**b**) relationship between the peak current and scan rate in logarithmic format; (**c**) capacitive contribution to charge storage at a scan rate of 1 mV s^−1^; (**d**) the contribution ratio of the capacitive and intercalated charge to capacity at different scan rates.

The electrochemical behavior of PPHC electrodes was investigated by cyclic voltammetry (CV) at 0.1 mV s^−1^ (Figure 6 and Figure S11). The reduction peak at around 0.5 V in the first cycle is related to SEI formation, which disappeared from the second cycle. A pair of sharp redox peaks was observed at ~0.0 V and ~0.1 V, corresponding to Na^+^ intercalation or nanopore filling. PPHC-900 displayed more pronounced capacitive Na^+^ storage in the range of 0.1–1.0 V, which matched with the capacity above 0.1 V in the charge–discharge curve. To further explore the sodium storage mechanism, CV curves were obtained at various scan rates (Figure 6b). The relationship between current *i* and scan rate *v* can be described using the equation *i* = *av^b^*, and the *b* value can be used to analyze the sodium storage behavior. When *b* = 1, the electrochemical mechanism is controlled by a capacitive process, which is related to Na^+^ adsorption on the surface active sites. If *b* = 0.5, it indicates a diffusion-controlled electrochemical process, signaling Na^+^ intercalation or nanopore filling [57]. All the PPHC samples presented a combined mechanism, including both capacitive and diffusion-controlled processes, as revealed by the *b* value between 0.6 and 0.7. PPHC-1300 had the lowest *b* value of 0.69, while PPHC-1100 showed the highest *b* value of 0.75. The Na^+^ adsorption-related capacitive-controlled capacity and the diffusive Na^+^ intercalation can be further quantitatively divided using the equation $i\left(v\right)=k_{1}v+k_{2}v^{1/2}$. PPHC-1100 presented a higher ratio of capacitive ion storage of 70% at 1 mV s^−1^, compared to 60% for PPHC-900 (Figure 6c and Figure S12). The capacitive-controlled electrochemical process also ensured the rate performance of PPHC-1100. The Nyquist plot of the impedance of the electrodes is shown in Figure 6d. The high-frequency semicircle region corresponds to the charge transfer resistance, and the low-frequency straight line is related to the diffusion or capacitive behavior of ions. PPHC-1100 and PPHC-1200 demonstrated lower charge transfer resistances of 50 Ω and 63 Ω, respectively, in contrast to 106 Ω for PPHC-900. Additionally, the two electrodes exhibited obvious capacitive behavior as indicated by the high degree of slope, whereas PPHC-900 presented a near 45° slope in the low-frequency region. Low charge transfer resistance and improved ion transport properties confer PPHC-1100 a high capacity and excellent rate capability.

**Figure 7:**
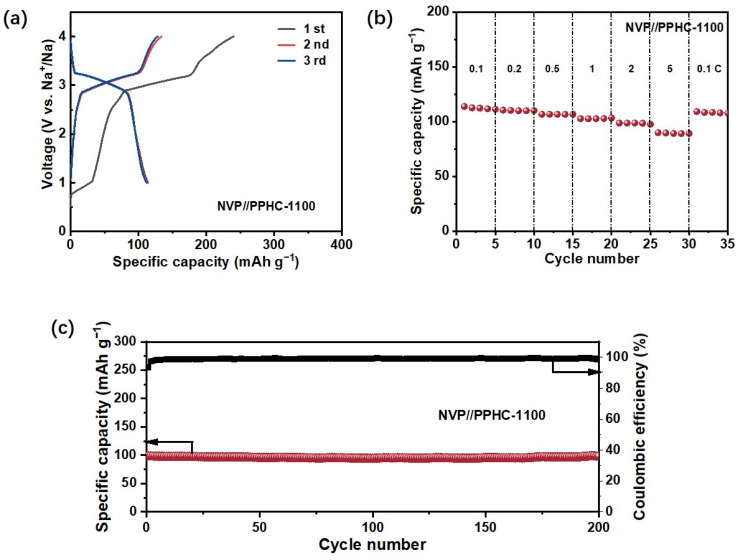
(**a**) The first three cycles of charge–discharge curves of NVP//PPHC-1100 at 0.1 C in the voltage range of 1–4 V; (**b**) rate performance of NVP//PPHC-1100 at rates from 0.1 to 5 C; (**c**) cycling performance at a current density of 0.5 C for NVP//PPHC-1100.

Given the significant reversible capacity, excellent rate performance, and cycling stability of PPHC-1100 observed in half cells, it was essential to further investigate its electrochemical behavior in full cells with a Na_3_V_2_(PO_4_)_3_ (NVP) cathode. Figure 7a shows that the full cell operates at a voltage range of 2.7–3.3 V, which meets the requirements for commercialization. The discharge specific capacity at 0.1 C (1 C = 200 mA g^−1^) was 112 mAh g^−1^ with an initial Coulombic efficiency (ICE) of 47.6%. At rates of 0.2, 0.5, 1, 2, and 5 C, the capacity can reach 110, 106, 102, 98, and 90 mAh g^−1^, respectively (Figure 7b). The capacity was then recovered to 108 mAh g^−1^ when the rate was lowered to 0.1 C. Additionally, after 200 cycles at 0.5 C, the NVP//PPHC-1100 full cell maintained a remarkable capacity retention over 95% (Figure 7c). These results indicate its strong potential for practical applications, offering a promising pathway for developing low-cost and long-life sodium-ion batteries (SIBs).

## 3. Materials and Methods

### 3.1. Materials Preparation

The pomegranate peel (PP) was derived from pomegranate products produced in Xi’an, Shaanxi Province, China. The PP was cut into pieces and washed thoroughly with deionized water and ethanol under ultrasonication. The pretreated PP was dried in a vacuum oven at 100 °C overnight. The pyrolysis of PP was conducted using a tube furnace at various temperatures of 900, 1000, 1100, 1200, and 1300 °C for 2 h, with a heating rate of 3 °C min^−1^ under argon protection. To remove inorganic impurities, the as-prepared raw carbon materials were subsequently washed with HCl (material:acid = 1:10) and HF (material:acid = 1:5) at 60 °C for 3 h. The obtained hard carbon was denoted as PPHC-900, PPHC-1000, PPHC-1100, PPHC-1200, and PPHC-1300. For comparison, the hard carbon carbonized at 1100 °C without acid post-treatment was obtained and named PPHC-N-1100.

### 3.2. Physicochemical Characterization

The crystalline structure of the materials was characterized by an X-ray diffractometer (XRD) (Bruker D8 Advance, Billerica, MA, USA) using a Cu Kα line (λ = 0.154 nm). Raman spectroscopy was used to examine the molecular and electronic structure of the carbon materials using a DXRxi Raman Microscope (Thermo Fisher Scientific, Waltham, MA, USA). The sample powder was mounted on a glass slide using double-sided adhesive tape. Raman spectra were acquired under a 532 nm laser (2.5 mW power) with 900 lines mm^−1^ grating. The sampling involved 100 exposures, each with an exposure time of 0.25 s. The morphology of the samples was observed by scanning electron microscopy (SEM, FEI Verios 460, FEI, Hillsboro, OR, U.S.) using an accelerating voltage of 2 kV (for the raw PPHC samples) and 5 kV (for PPHC-1100 electrode before and after cycling). The local microstructure and microcrystalline status of the materials were studied by transmission electron microscopy (TEM, FEI Tecnai G2 F20, FEI, Hillsboro, OR, USA) using an accelerating voltage of 200 kV. X-ray photoelectron spectroscopy (XPS) was performed using an X-ray photoelectron spectrometer (PerkinElmer PHI 1600 ESCA, PerkinElmer, Waltham, MA, USA). The porosity status of the samples was detected and analyzed by a surface area and pore size analyzer (BET, Micromeritics ASAP 2460, Micromeritics, Norcross, GA, USA) via N_2_ adsorption/desorption tests.

### 3.3. Electrochemical Study

The hard carbon materials were mixed with a polymer binder, polyvinylidene difluoride (PVDF), and carbon black (Ketjenblack EC-600JD, Nouryon, Osaka, Japan) in a weight ratio of 8:1:1 in N-methyl-2-pyrrolidone (NMP) solvent to form a slurry. The slurry was then coated onto an Al foil by doctor-blading to obtain the battery electrode. The cathode Na_3_V_2_(PO_4_)_3_ (NVP) was prepared using a similar method. Coin-type cells were assembled in an argon-filled glove box using the carbon electrode, sodium foil, and a glass fiber separator. The electrolyte was 1 M NaClO_4_ in a mixed solution of dimethyl carbonate (DMC) and ethylene carbonate (EC) (volume ratio 1:1). The cell was then used for electrochemical performance evaluation and electrochemistry behavior investigation. Galvanostatic charging/discharging tests were performed on a battery testing system (Neware CT-4000, Neware Technology Ltd., Shenzhen, China) with a voltage range of 0.001–2.5 V. Cyclic voltammetry (CV) and electrochemical impedance spectroscopy (EIS) tests were conducted on an electrochemical workstation (Chenhua CHI660E, Shanghai Chenhua Instrument Co., Ltd., Shanghai, China). The scanning rate of CV was 0.1 mV s^−1^, and the voltage range was 0.01–2.5 V. Electrochemical impedance (EIS) was tested in the frequency range of 0.01–100,000 Hz at an amplitude of 5 mV.

## 4. Conclusions

In conclusion, a novel hard carbon material (PPHC) was prepared by direct pyrolysis of pomegranate peel at various temperatures. XRD, Raman, XPS, and electron microscopy revealed the structure evolution of PPHC with carbonization temperature. The interlayer spacing of the PPHC increased with temperature and then decreased when the temperature exceeded 1300 °C. The PPHC obtained at 1100 °C had an expanded interlayer spacing of 0.38 nm, an appropriate oxygen content, and a low surface specific area. PPHC-1100 presented excellent electrochemical performance as an anode in sodium-ion batteries, exhibiting both the highest slope capacity and plateau capacity. It delivered a high reversible capacity of 330 mAh g^−1^ at 0.1 C and maintained a capacity of 175 mAh g^−1^ at 0.5 C after 200 cycles. The appropriate interlayer spacing guaranteed the plateau capacity, while the improved ion transport ability ensured the slope capacity as well as rate performance. This research developed a novel biomass-derived hard carbon for sodium-ion battery anodes and also provided a new strategy for the recycling of pomegranate waste. Derived from renewable, low-cost biowaste, pomegranate peel carbon can achieve tunable structures through appropriate processing techniques. In addition to sodium-ion batteries, these materials also have potential applications in other energy storage areas, environmental remediation, and electrocatalysis.

## Data Availability

The data presented in this study are available on request.

## Supplementary Materials

The following supporting information can be downloaded at https://www.mdpi.com/article/10.3390/molecules29194639/s1. Figure S1. Fit components for all PPHC samples: PPHC-900 (a), PPHC-1000 (b), PPHC-1100 (c), PPHC-1200 (d) and PPHC-1300 (e), PPHC-N-1100 (f). Figure S2: The XPS survey spectra of all PPHC samples (dark gray: PPHC-900, red: PPHC-1000, blue: PPHC-1100, green: PPHC-1200, purple: PPHC-1300); Figure S3: The XPS C1s spectra of PPHC-900 (a), PPHC-1000 (b), PPHC-1200 (c), and PPHC-1300 (d); Figure S4: The XPS O1s spectra of PPHC-900 (a), PPHC-1000 (b), PPHC-1200 (c), and PPHC-1300 (d); Figure S5: SEM images of PPHC-1000 (a), PPHC-1200 (c); TEM images and corresponding interlayer spacing of PPHC-1000 (b), PPHC-1200 (d) (inset: SAED pattern); Figure S6: The SEM image of PPHC-N-1100 (a) and TEM image of PPHC-N-1100 (b); Figure S7. N_2_ adsorption–desorption isotherms (a) and pore size distributions (b–g) of all samples. Figure S8: The first three charge–discharge cycles of PPHC-1000 (a), PPHC-1200 (b), PPHC-N-1100 (c) obtained at 0.1 C; Figure S9. Charge discharge at different rate for all PPHC samples. Figure S10. SEM images of PPHC-1100 before (a) and after (b) 100 charge-discharge cycles at a current density of 100 mA/g. Figure S11: CV curves of PPHC-900 (a), PPHC-1000 (b), PPHC-1200 (c), and PPHC-1300 (d) at the scan rate of 0.1 mV s^−1^; Figure S12: CV curves of PPHC-900 at the scan rate of 0.1 mV s^−1^ (a), and the capacitive contribution to charge storage at a scan rate of 0.2 mV s^−1^ (b), 0.4 mV s^−1^ (c), 0.6 mV s^−1^ (d), 0.8 mV s^−1^ (e), 1 mV s^−1^ (f). Table S1. Comparisons of the electrochemical performance of different hard carbon anodes for SIBs. Refs. [58,59,60,61,62,63] are cited in the Supplementary Materials.
