# Supplementary material for: Pomegranate Peel-Derived Hard Carbons as Anode Materials for Sodium-Ion Batteries
Source: Molecules. 2024 Sep 29;29(19):4639. doi: 10.3390/molecules29194639 (PMC11478217; doi:10.3390/molecules29194639)
Supplement: Supplementary file 1 [file molecules-29-04639-s001.zip › molecules-3152194-supplementary.pdf]

Supporting Information

Pomegranate Peel-Derived Hard Carbons as Anode Materials for Sodium-Ion Batteries

Qijie Wu [1], Kewei Shu [2,*], Long Zhao [1] and Jianming Zhang [1,*]

[1] Institute of Quantum and Sustainable Technology (IQST), School of Chemistry and Chemical Engineering, Jiangsu University, Zhenjiang 212013, China

[2] College of Chemistry and Chemical Engineering, Shaanxi University of Science and Technology, Xi'an 710021, China

* Correspondence: shukw@sust.edu.cn (K.S.); zhangjm@ujs.edu.cn (J.Z.)

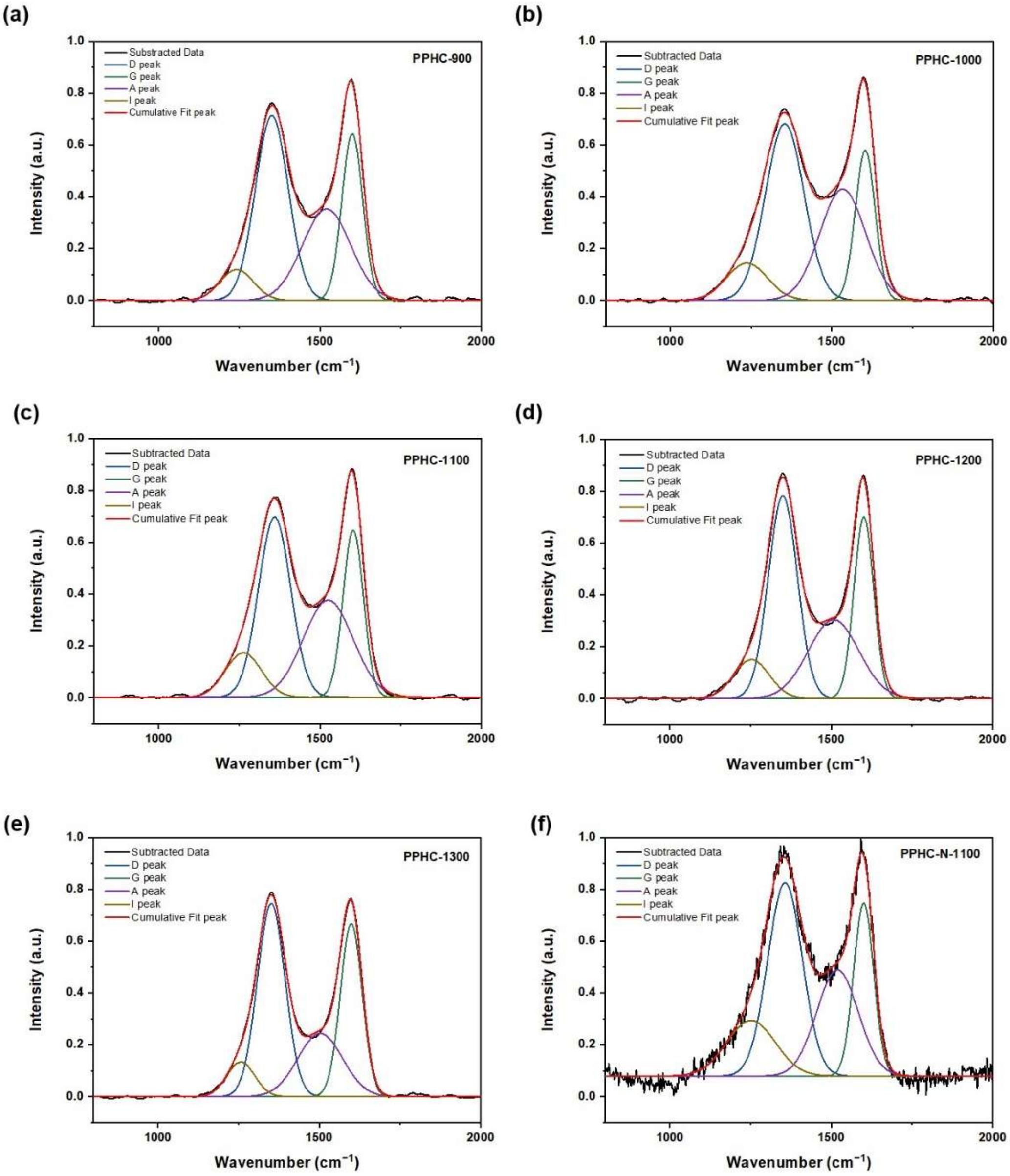

Figure S1. Fit components for all PPHC samples: PPHC-900 **(a)**, PPHC-1000 **(b)**, PPHC-1100 **(c)**, PPHC-1200 **(d)** and PPHC-1300 **(e)**, PPHC-N-1100 **(f)**.

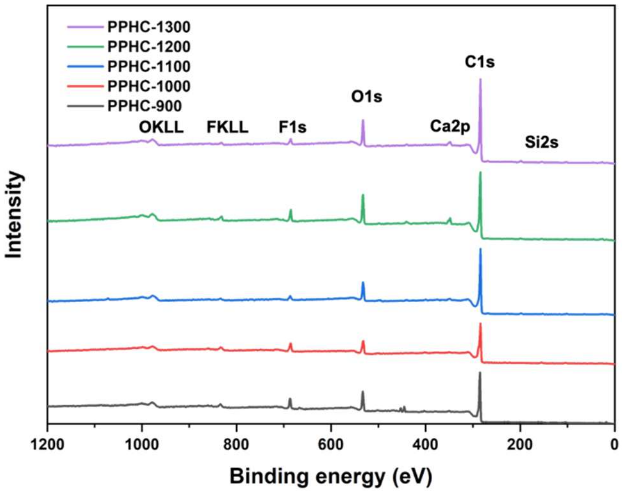

Figure S2. The XPS survey spectra of all PPHC samples (dark gray: PPHC-900, red: PPHC-1000, blue: PPHC-1100, green: PPHC-1200, purple: PPHC-1300).

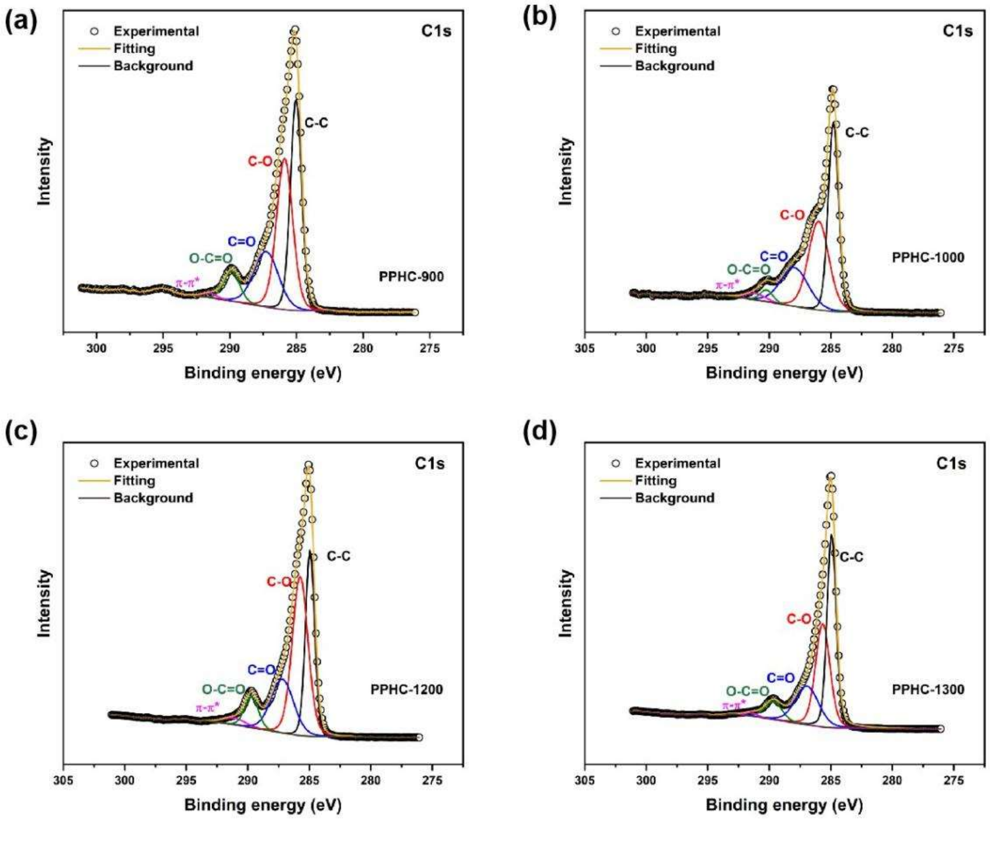

Figure S3. The XPS C1s spectra of PPHC-900 **(a)**, PPHC-1000 **(b)**, PPHC-1200 **(c)**, and PPHC-1300 **(d)**.

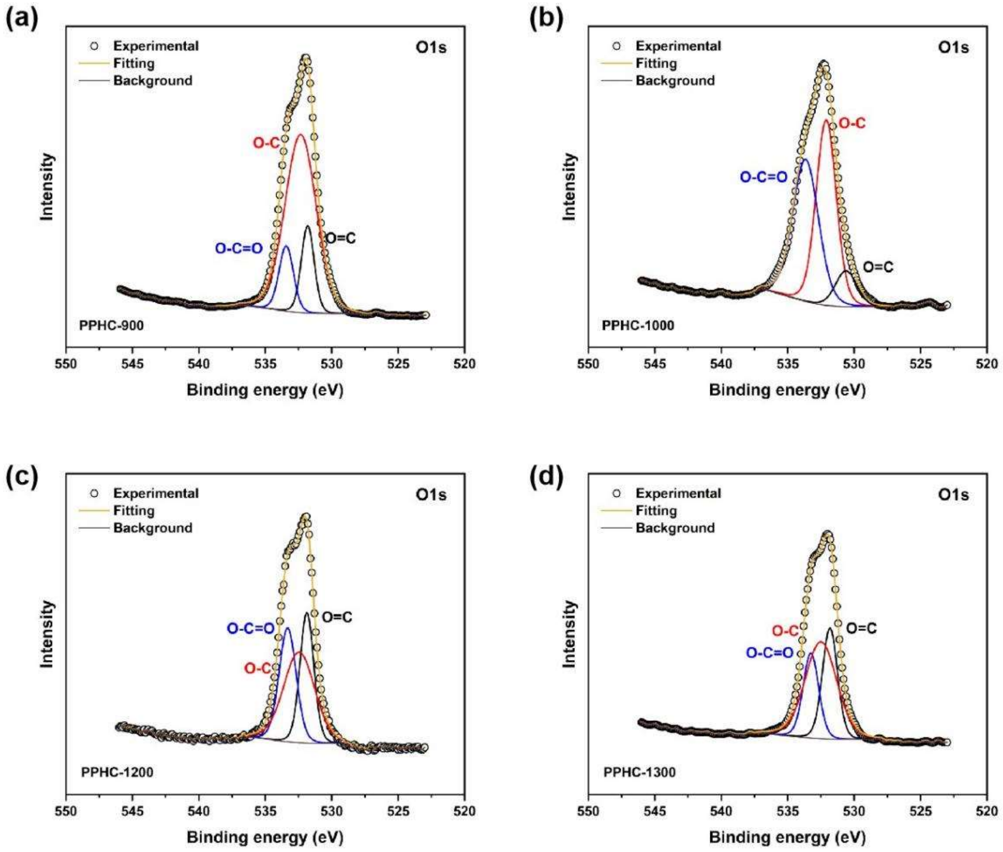

Figure S4. The XPS O1s spectra of PPHC-900 **(a)**, PPHC-1000 **(b)**, PPHC-1200 **(c)**, and PPHC-1300 **(d)**.

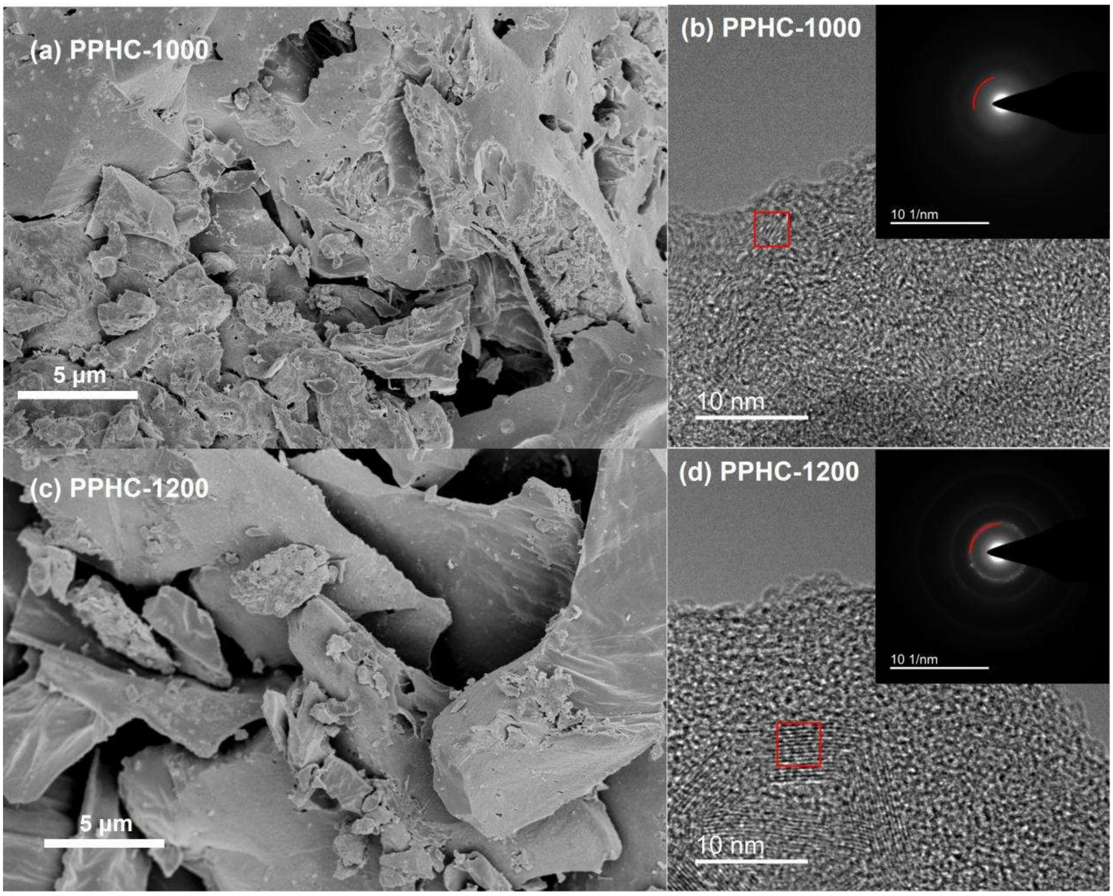

Figure S5. SEM images of PPHC-1000 **(a)**, PPHC-1200 **(c)**; TEM images of PPHC-1000 **(b)**, PPHC-1200 **(d)** (inset: SAED pattern).

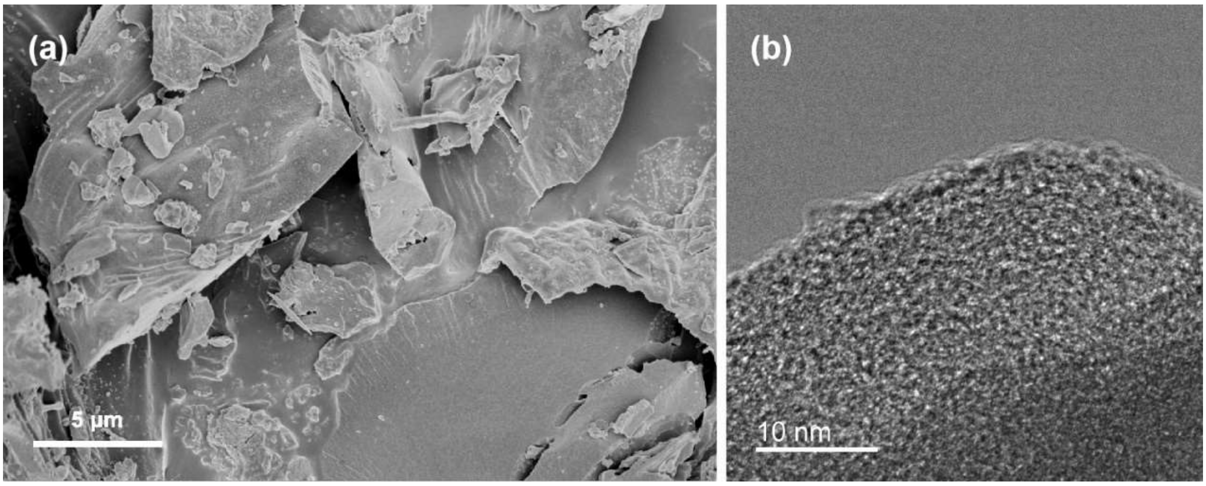

Figure S6. The SEM image of PPHC-N-1100 **(a)** and TEM image of PPHC-N-1100 **(b)**.

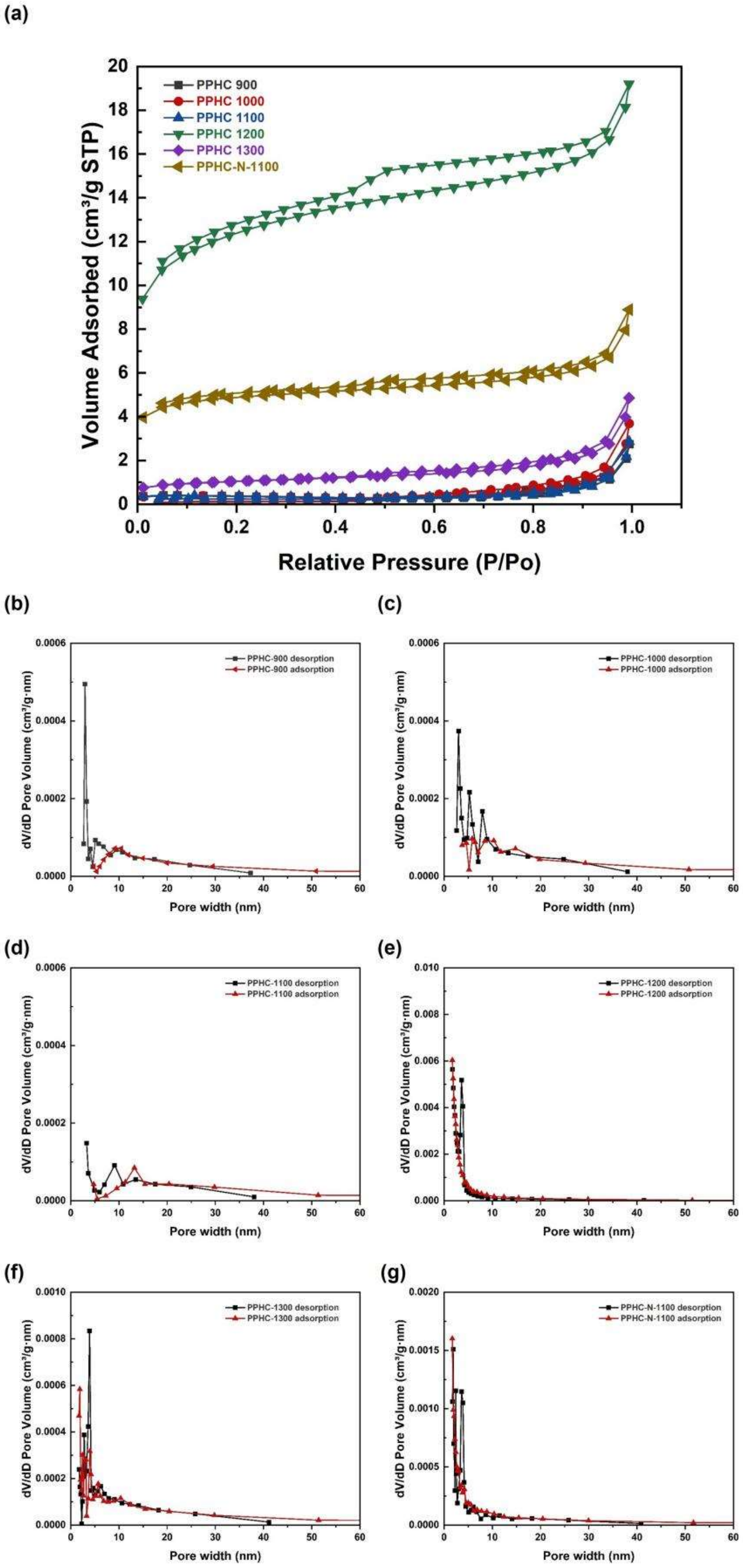

Figure S7. N_2 adsorption－desorption isotherms **(a)** and pore size distributions **(b-g)** of all samples

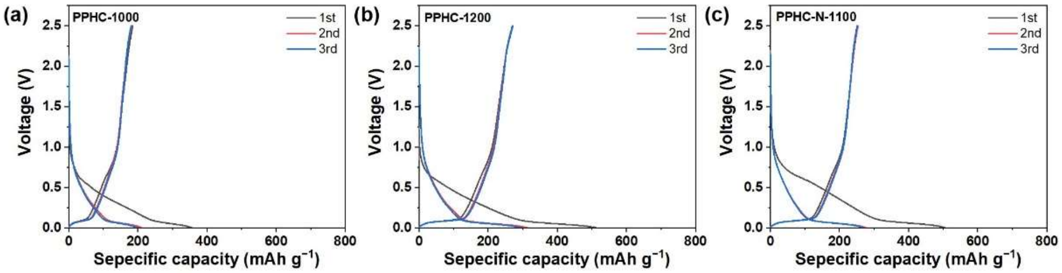

Figure S8. The first three charge discharge cycles of PPHC-1000 **(a)**, PPHC-1200 **(b)**, PPHC-N-1100 **(c)** obtained at 0.1 C.

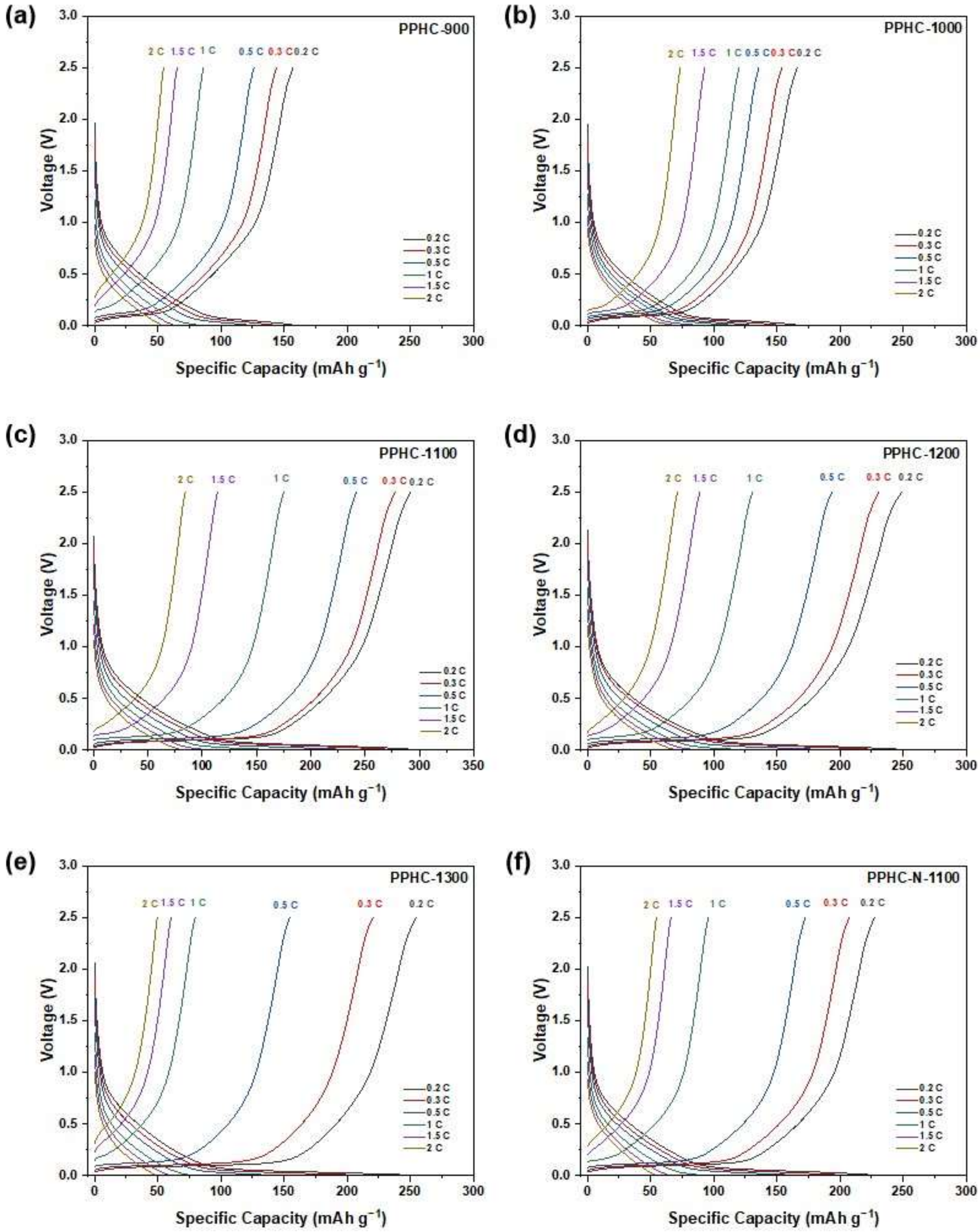

Figure S9. Charge discharge at different rate for all PPHC samples.

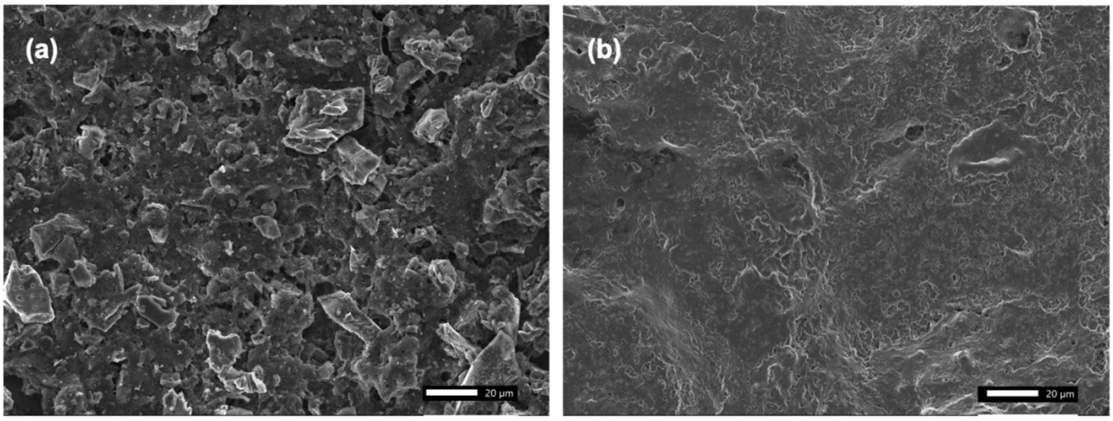

Figure S10. SEM images of PPHC-1100 before **(a)** and after **(b)** 100 charge-discharge cycles at a current density of 100 mA/g.

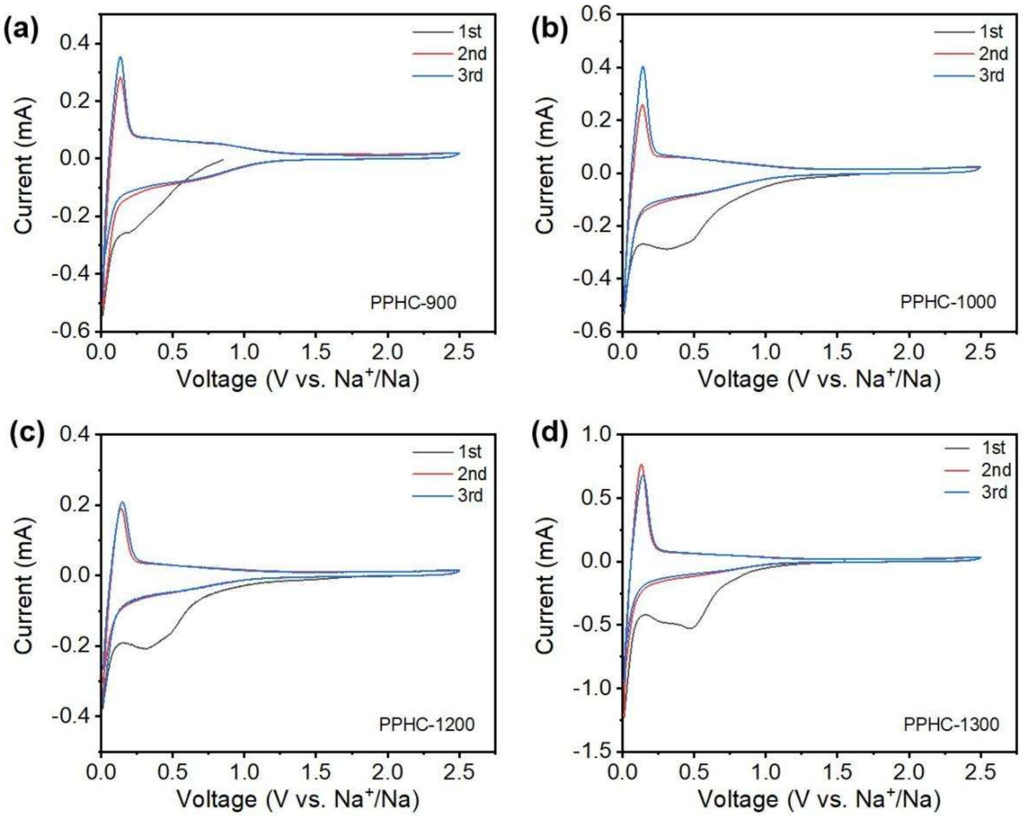

Figure S11. CV curves of PPHC-900 **(a)**, PPHC-1000 **(b)**, PPHC-1200 **(c)**, and PPHC-1300 **(d)** at the scan rate of 0.1 mV s^{-1}.

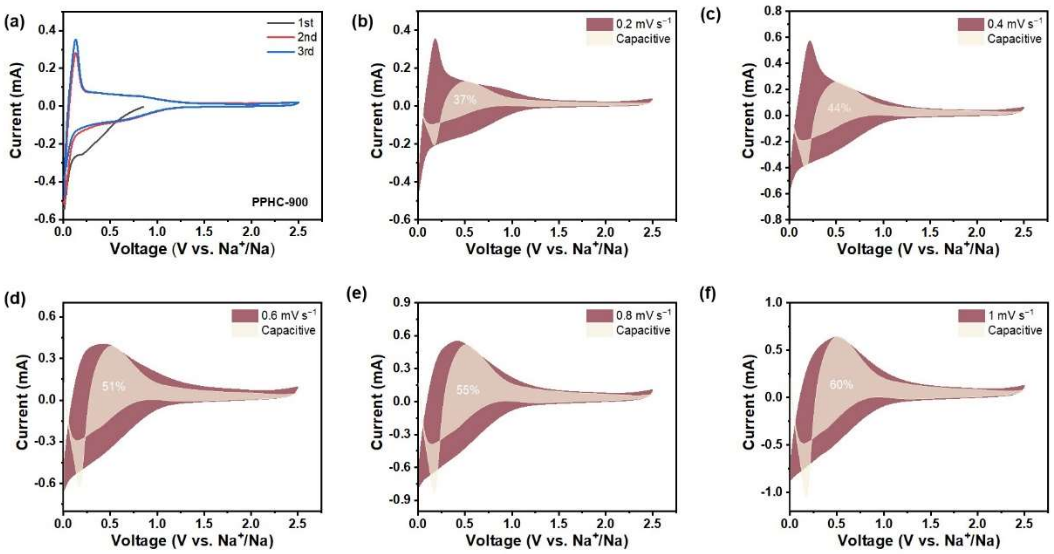

Figure S12. CV curves of PPHC-900 at the scan rate of 0.1 mV s^{-1} **(a)**, and the capacitive contribution to charge storage at a scan rate of 0.2 mV s^{-1} **(b)**, 0.4 mV s^{-1} **(c)**, 0.6 mV s^{-1} **(d)**, 0.8 mV s^{-1} **(e)**, 1 mV s^{-1} **(f)**.

Table S1. Comparisons of the electrochemical performance of different hard carbon anodes for SIBs.

**Precursors**	**Preparation conditions**	**Reversible capacity (mAh/g)**	**Current Density**	**ICE (%)**	**Ref.**
Golden Berry Leaves	Carbonized at 1400°C	338.7	20 mA/g	86.43	[22]
Apple Biowaste	Phosphoric acid activation, pyrolyzed at 1100°C	245	0.1 C	61	[58]
Banana Peels	Pyrolysis at 1100°C	336	100 mA/g	67.8	[59]
Indonesian snake fruit peel	Pre-carbonization at 800°C, KOH activation	255	0.1 A/g	38~42	[60]
Aegle marmelos shell	Hydrothermal, calcination at 900 °C	223	10 mA/g	76	[61]
Rambutan Peel	Hydrothermal, pyrolysis at 1000 °C	225	0.1 A/g	55	[62]
Date palm Seed	Pyrolyzed at 1400 °C	274	25 mA/g	88.4	[63]
Rice-husk	Pyrolyzed at 1300 °C	372	25 mA/g	66	[21]
corncob	Carbonized at 950 °C	264.3	1 C	59.4	[23]
This work	pyrolysis at 1100 °C, acid-treatment	330	0.1 C	44.8	
